# Serial enumeration of circulating tumor cells predicts treatment response and prognosis in metastatic breast cancer: a prospective study in 393 patients

**DOI:** 10.1186/1471-2407-14-512

**Published:** 2014-07-11

**Authors:** Markus Wallwiener, Sabine Riethdorf, Andreas Daniel Hartkopf, Caroline Modugno, Juliane Nees, Dharanija Madhavan, Martin Ronald Sprick, Sarah Schott, Christoph Domschke, Irène Baccelli, Birgitt Schönfisch, Barbara Burwinkel, Frederik Marmé, Jörg Heil, Christof Sohn, Klaus Pantel, Andreas Trumpp, Andreas Schneeweiss

**Affiliations:** 1 National Center for Tumor Diseases, Im Neuenheimer Feld 460, 69120 Heidelberg, Germany; 2 Department of Obstetrics and Gynecology, University of Heidelberg, Im Neuenheimer Feld 440, 69120 Heidelberg, Germany; 3 Department of Tumor Biology, University Medical Center Hamburg-Eppendorf, Martinistraße 52, 20246 Hamburg, Germany; 4 Department of Obstetrics and Gynecology, University of Tübingen, Calwerstraße 7, 72076 Tübingen, Germany; 5 Division of Stem Cells and Cancer, German Cancer Research Center (DKFZ), Im Neuenheimer Feld 280, 69120 Heidelberg, Germany; 6 Heidelberg Institute for Stem Cell Technology and Experimental Medicine (HI-STEM gGMBH), Im Neuenheimer Feld 280, 69120 Heidelberg, Germany

**Keywords:** Metastatic breast cancer, Circulating tumor cells, Systemic therapy, Treatment response, Survival

## Abstract

**Background:**

To prospectively assess circulating tumor cell (CTC) status at baseline (CTC_BL_) and after one cycle of a new line of systemic therapy (CTC_1C_), and changes from CTC_BL_ to CTC_1C_ (CTC kinetics, CTC_KIN_) for their utility in predicting response, progression-free (PFS) and overall survival (OS) in metastatic breast cancer (MBC).

**Methods:**

CTC_BL_ and CTC_1C_ status was determined as negative (-) or positive (+) for < 5 or ≥ 5 CTCs/7.5 ml blood using CellSearch™ (Veridex). CTC_KIN_ was categorized as favorable (CTC_1C_-) or unfavorable (CTC_1C_+). Tumor response was to be assessed every 2–3 months using the Response Evaluation Criteria in Solid Tumors (RECIST) criteria. Statistical analysis focused on the relation between CTC status and CTC_KIN_, and response, PFS, and OS.

**Results:**

133/393 (34%) patients enrolled were CTC_BL_+. CTC_1C_ status after one cycle and radiological tumor response were assessed after median (range) periods of 1.2 (0.5–3.2) and 2.9 (0.5–4.8) months, respectively. 57/201 (28%) were CTC_1C_+. Median [95% confidence interval] PFS and OS (months) were significantly reduced in CTC_BL_+ vs. CTC_BL_- patients (PFS 4.7 [3.7–6.1] vs. 7.8 [6.4–9.2]; OS 10.4 [7.9–15.0] vs. 27.2 [22.3–29.9]), and for CTC_1C_+ vs. CTC_1C_- patients (PFS 4.3 [3.6–6.0] vs. 8.5 [6.6–10.4]; OS 7.7 [6.4–13.9] vs. 30.6 [22.6–not available]). Unfavorable CTC_KIN_ was significantly associated with progressive disease. Multivariate Cox regression analysis revealed prognostic factors for shorter PFS (CTC_BL_+, persistent CTCs after one cycle, ≥ 3rd-line therapy, and triple-negative receptor status) and shorter OS (CTC_BL_+, persistent CTCs after one cycle, bone-and-visceral/local metastases, ≥ 3rd-line therapy, and triple-negative receptor status).

**Conclusions:**

CTC_BL_, CTC_1C_, and CTC_KIN_ are predictive of outcome in MBC. Serial CTC enumeration is useful in tailoring systemic treatment of MBC.

**Trial registration:**

Not applicable.

## Background

Apart from the expression of hormone and human epidermal growth factor receptors there are as yet hardly any predictive factors for treatment efficacy in patients with metastatic breast cancer (MBC) despite a rapidly growing number of treatment options. In this situation it is of utmost importance to identify early indicators of response to systemic treatment to avoid unnecessary exposure to ineffective but toxic therapies and to enable prognostication of progression-free survival (PFS) and overall survival (OS). Circulating tumor cells (CTCs) have been detected in 40–60% of patients with MBC using the CellSearch™ system (Veridex) [1,2]. The presence of CTCs at levels ≥ 5/7.5 ml peripheral blood is associated with decreased PFS and OS [2-4]. It has been suggested that CTCs provide more clinically relevant information than conventional imaging studies regarding therapeutic efficacy and ultimate outcome [5]. In addition, the prognostic information of ≥ 5 CTCs/7.5 ml blood might be helpful in identifying those patients who would likely experience a worse outcome with standard treatment and might benefit from more aggressive therapy [4]. Thus far, several retrospective and a few prospective studies in patients with MBC have demonstrated the usefulness of monitoring therapeutic efficacy by serial CTC enumerations [6-9]. To further address this important issue, the present study aimed to prospectively assess in a large group of patients whether CTC status at baseline (CTC_BL_) and after one cycle of a new line of treatment (CTC_1C_) and changes in CTC status from baseline to completion of one treatment cycle (CTC kinetics, CTC_KIN_) could serve as early predictors of efficacy in terms of response, PFS, and OS.

## Methods

### Patients and study design

This was a prospective, single-center, non-randomized, partially blinded, treatment-based study. The study was blinded in the following respects. Both patients and treating physicians were blinded to CTC status, and hence treatment regimens did not depend on CTC status. All investigators and technical staff who performed or reviewed the CTC studies were blinded to patient history and treatment. CTC enumeration and characterization were confirmed by independent reviewers. All radiologists performing computed tomography (CT) scans and magnetic resonance imaging (MRI) studies were blinded to the patient’s treatment regimen. The study was conducted at the National Center for Tumor Diseases (NCT), Heidelberg, Germany and the Department of Obstetrics and Gynecology, University of Heidelberg, Heidelberg, Germany.

Patients included in the study were women with MBC about to start a new line of systemic treatment. Patients were enrolled consecutively between March 2010 and December 2013. Main eligibility criteria were clinical and radiological evidence of measurable or evaluable metastatic disease according to the Response Evaluation Criteria in Solid Tumors (RECIST) criteria [10], age > 18 years, progressive metastatic disease, CTC assessment at baseline, and written informed consent. Before starting a new line of systemic treatment, patients underwent CTC enumeration to determine CTC_BL_ status, defined as positive (CTC_BL_+) for ≥ 5 CTC or negative (CTC_BL_-) for < 5 CTC per 7.5 ml of peripheral blood [11]. Determination of CTC status was repeated after the first cycle of treatment (CTC_1C_). After approx. 3 months, patients were evaluated for response by CT and MRI, as appropriate. Response was defined as complete response (CR), partial response (PR), stable disease (SD), and progressive disease (PD) according to the RECIST criteria, version 1.1 [10]. Evaluation was repeated according to the RECIST criteria every 2–3 months until progression of disease. Survival status was recorded until death or loss to follow-up.

All study procedures, including laboratory evaluations, imaging studies, and treatment planning, were carried out at the NCT, Heidelberg, Germany and the Department of Obstetrics and Gynecology of the University of Heidelberg, Heidelberg, Germany in collaboration with the German Cancer Research Center (DKFZ), Heidelberg, Germany, the Department of Tumor Biology, University Medical Center Hamburg-Eppendorf, Hamburg, Germany, and the Heidelberg Institute for Stem Cell Technology and Experimental Medicine (HI-STEM), Heidelberg, Germany. Ethical approval was obtained from the Ethics Committee of the Medical Faculty of the University of Heidelberg.

### CTC enumeration

For CTC enumeration, 7.5 ml peripheral whole blood was collected in a standard 10-ml tube containing ethylenediaminetetraacetic acid (EDTA) and a cellular preservative. Blood samples were kept at room temperature for ≤ 72 hours before analysis using the CellSearch™ assay (CellSearch™ Epithelial Cell Kit/CellSpotter™ Analyzer, Veridex LLC, Raritan, NJ, USA). Sample processing and analysis were done strictly according to the manufacturer’s instructions. The assay uses a ferrofluid coated with antibodies to epithelial cell adhesion molecule (EpCAM) to immunomagnetically separate cells of epithelial origin from blood, and fluorescent staining to differentiate between debris, hematopoietic cells, and epithelial-derived circulating tumor cells [12]. It provides high intra-observer, inter-observer and inter-instrument agreement [2,13]. Thus, CTCs enumerated and characterized in this study were cells with positive nuclear staining expressing cytokeratin (CK) 8, 18, and 19, and lacking CD45 [11,14]. Assay operators were trained to classify images generated by the CellSpotter™ Analyzer before study initiation. Samples with < 5 CTCs/7.5 ml were classified as CTC-, those with ≥ 5 CTCs/7.5 ml as CTC+ [11]. CTC kinetics (CTC_KIN_) were defined in terms of changes in CTC status from CTC_BL_ to CTC_1C_ and categorized as favorable (CTC_BL_- to CTC_1C_- and CTC_BL_+ to CTC_1C_-) or unfavorable (CTC_BL_- to CTC_1C_+ and CTC_BL_+ to CTC_1C_+).

### HER2 status

Human epidermal growth factor receptor 2 (HER2) status was determined using the immunohistochemistry-based HERCEP™ test (DAKO, Glostrup, Denmark) for semi-quantitative detection of HER2 expression in breast cancer tissue. Expression of HER2 was scored on a scale from 0 to 3+. Tissue samples with a score of 3+ were considered HER2-positive. Whenever the score was 2+, HER2 amplification was determined by fluorescence in-situ hybridization using the Pathvysion Kit (Vysis Inc., Downers Grove, IL, USA).

### Data analysis and statistics

Patient demographic and clinical characteristics were summarized as medians and ranges or numbers and percentages, as appropriate. The numbers of missing values were given in ‘no data’ categories. Differences between the CTC+ and CTC- groups were compared using the Wilcoxon rank test and Fisher’s exact test, as appropriate. PFS was defined from date of enrollment until the date of disease progression or death from any cause, whichever occurred first. OS was calculated from the date of enrollment until the date of death from any cause. Patients who were alive or showed no progression at last follow-up were regarded as censored observations. Median follow-up time was calculated using the reverse Kaplan-Meier method.

To identify predictors of PFS and OS, the following candidate predictors were selected a priori based on previous studies and univariate analysis: CTC_BL_ status (negative or positive), age at study entry, molecular subtypes (hormone receptor (HR)+/HER2-, HER2+, or triple negative breast cancer (TNBC)), site of metastasis (local, bone/visceral, or both), number of metastatic sites (one or at least two), and line of therapy (first, second, or at least third). The prognostic effects of these factors were determined by multivariate analysis using a Cox proportional hazards regression model. Patients with missing values in these variables were not included in the Cox regression models. Separate models for CTC_BL_ and CTC_KIN_ were formulated because the CTC_BL_ model showed a fairly larger sample size and to avoid multicollinearity (since CTC_BL_ and CTC_KIN_ are related). Concordance indices were used to estimate the predictive accuracy of the Cox models.

During the initial phase of the study, which comprised the first 100 patients, CTC_1C_ status was routinely determined only in CTC_BL_+ patients and not in CTC_BL_- patients. However, as preliminary CTC_1C_ results from CTC_BL_- patients also drew interest, it was decided to determine CTC_1C_ status in all subsequent patients. This change may have introduced a potential source of bias in the CTC_1C_ results, e.g. proportions. All CTC_KIN_ findings were, thus, conditioned on survival up to the determination of CTC_1C_ status.

Statistical analyses were performed using R (version 3.0.0, package *survival*). All reported *P* values were two-sided and a significance level of 5% was chosen.

## Results

### Patients and study design

**Figure 1:**
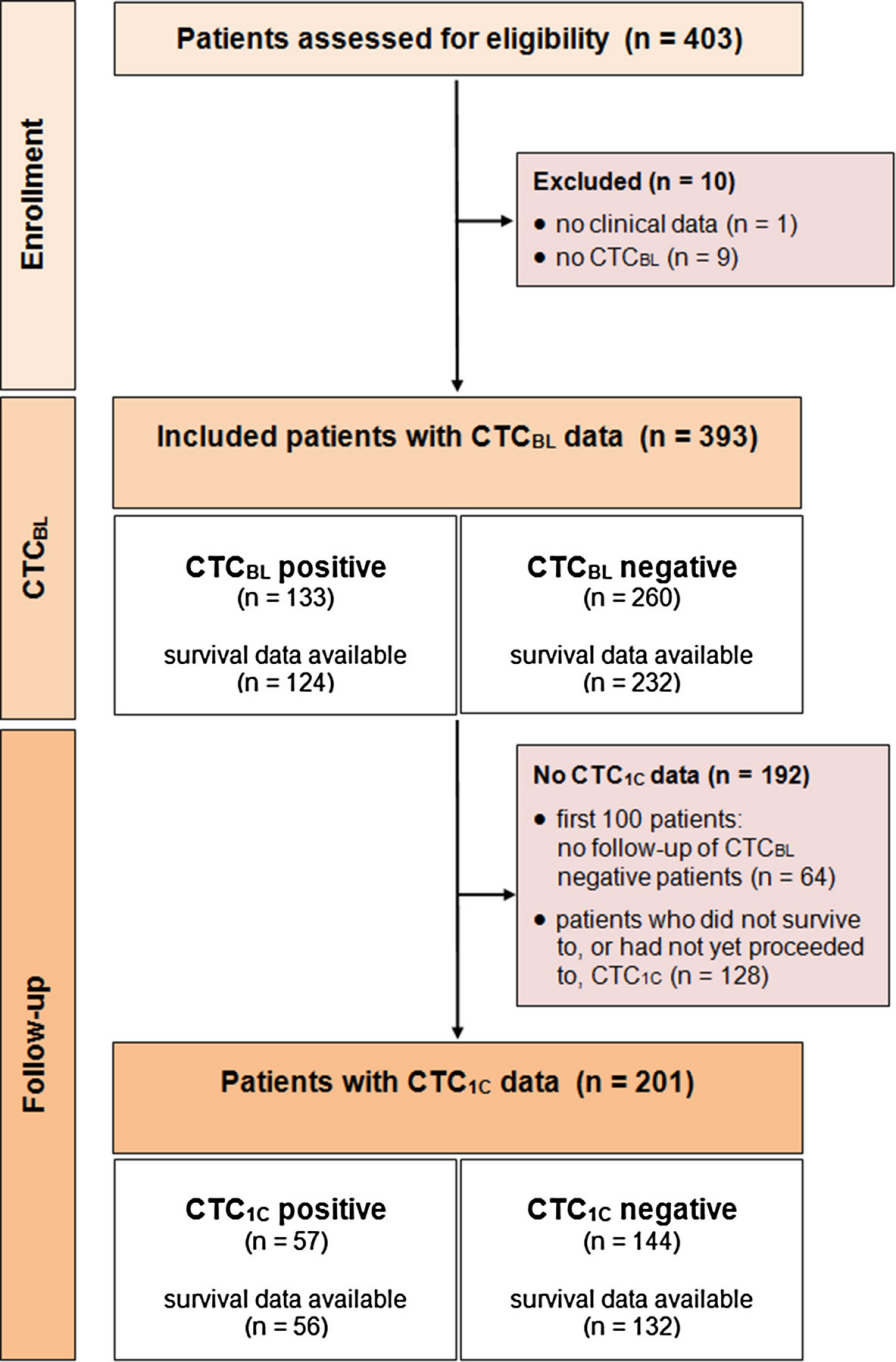
**Flow of patients through the study.** Of 403 consecutive patients assessed for eligibility, 10 (2.5%) were excluded from the study because essential data items were not available (no clinical data: 1 patient; no CTC_BL_ data: 9 patients). Of the 393 patients included in the study, 192 had no CTC_1C_ counts and were therefore excluded from further analysis for the following reasons. During the initial phase of the study, i.e. the first 100 patients, CTC_1C_ status was routinely determined only in CTC_BL_+ patients, resulting in 64 CTC_1C_- patients without CTC_1C_ counts. Of the remaining 128 patients without CTC_1C_ counts, 12 were excluded because blood samples were not obtained within the predefined study timeframe of 0.5–3.2 months, 25 did not survive to CTC_1C_ assessment because they died within the first 3.2 months, and 91 patients who survived beyond 3.2 months after inclusion had no CTC_1C_ count (41 had not yet proceeded to CTC_1C_ and 50 were lost to follow-up blood sampling as our center often treats external patients).

From March 2010 through December 2013, 403 consecutive patients were enrolled in the study. Figure 1 shows the flow of patients through the study. Reasons for exclusion from, or non-availability for, further analysis are detailed in the figure legend. Of the 393 evaluable patients with CTC_BL_ counts, 133 (34%) were CTC_BL_+ and 260 (66%) were CTC_BL_-. The two patient groups did not differ significantly in median age (range) at initial diagnosis of breast cancer (50 (28–81) vs. 51 (23–79) years) but age at study entry was significantly lower in CTC_BL_+ patients (57 (33–81) vs. 61 (29–89) years). Patient characteristics at baseline and after one cycle of treatment are summarized in Table 1. Notably, the majority of patients had ER+ (271/378 (72%)), PgR+ (240/370 (65%)), and HER2- (274/341 (80%)) primary tumors. Most patients had more than one metastatic site (305/393 (78%)) and approximately half of patients had both bone and visceral/local metastases (191/393 (49%)). At study entry, 135/391 (35%) patients were about to start third- or higher-line treatment.

### CTC status and response

CTC_1C_ status was assessed after a median (range) of 1.2 (0.5–3.2) months. CTC_1C_ status was positive in 57/201 (28%) and negative in 144/201 (72%) of patients. During the initial phase of the study, which comprised the first 100 patients, CTC_1C_ status was determined only in CTC_BL_+ patients. As shown in Table 1, at least SD (i.e. CR, PR, or SD) was seen in 162/255 (64%) patients at the 3-month radiological examination, of whom 52/162 (32%) were CTC_BL_+ while 110/162 (68%) were CTC_BL_-. Radiological restaging was performed a median of 2.9 (0.5–4.8) months after study entry. PD occurred in 93/255 (36%) patients, of whom 40/93 (43%) were CTC_BL_+ while 53/93 (57%) were CTC_BL_- (Fisher’ exact test, *P* = 0.104). CTC_KIN_ could be determined in 201 patients as both their CTC_BL_ and CTC_1C_ data were available. At least SD was achieved in 55/75 (73%) patients with CTC_KIN_ from CTC_BL_- to CTC_1C_-, 21/32 (66%) with CTC_KIN_ from CTC_BL_+ to CTC_1C_-, 20/41 (49%) with CTC_BL_+ to CTC_1C_+, and 3/6 (50%) with CTC_BL_- to CTC_1C_+ (Fisher’s exact test, *P* = 0.04997).

**Table 1:** Patient characteristics by CTC+ status at baseline (BL) and after one cycle of treatment (1C)

	**All patients, BL**	**CTC**_**BL**_**+**	***P***	**All patients, 1C**	**CTC**_**1C**_**+**	***P***
Patients	393	133 (34%)*		201	57 (28%)	
Age, median (range); years						
at initial diagnosis	51 (23–81)	50 (28–81)	0.853	50 (28–77)	50 (33–77)	0.570
at study inclusion	59 (29–89)	57 (33–81)	**0.030**	57 (33–89)	55 (33–77)	0.092
Baseline CTC count, median (range); number/7.5 ml blood	1 (0–930)	21 (5–930)		—	—	
ER status			0.631			0.729
ER+	271	94 (35%)		136	41 (30%)	
ER-	107	34 (32%)		55	15 (27%)	
No data	15	5 (33%)		10	1 (10%)	
PgR Status			0.819			0.866
PgR+	240	81 (34%)		124	36 (29%)	
PgR-	130	46 (35%)		64	20 (31%)	
No data	23	6 (26%)		13	1 (8%)	
HER2 status of primary tumor			0.119			**0.028**
HER2+	67	18 (27%)		30	4 (13%)	
HER2-	274	102 (37%)		142	49 (35%)	
No data	52	13 (25%)		29	4 (14%)	
Molecular subtypes			0.221			0.062
HR+/HER2-	216	83 (38%)		110	39 (35%)	
HER2+	67	18 (27%)		30	4 (13%)	
TNBC	57	19 (33%)		32	10 (31%)	
No data	53	13 (25%)		29	4 (14%)	
Metastasis site			**< 0.001**			**0.005**
Bone	68	25 (37%)		40	16 (40%)	
Visceral/local	134	28 (21%)		68	10 (15%)	
Both	191	80 (42%)		93	31 (33%)	
No data	0	0 (0%)		0	0 (0%)	
Number of metastasis sites			0.372			1.000
1	88	26 (30%)		48	14 (29%)	
≥ 2	305	107 (35%)		153	43 (28%)	
No data	0	0 (0%)		0	0 (0%)	
Line of therapy			0.724			0.097
1	175	62 (35%)		97	26 (27%)	
2	81	28 (35%)		44	8 (18%)	
≥ 3	135	42 (31%)		59	22 (37%)	
No data	2	1 (50%)		1	1 (100%)	
Treatments before study						
Hormonal therapy			0.904			0.167
Yes	289	97 (34%)		143	45 (31%)	
No	104	36 (35%)		58	12 (21%)	
No data	0	0 (0%)		0	0 (0%)	
Antibody therapy (bevacizumab or other)			0.210			**0.023**
Yes	103	47 (46%)		54	22 (41%)	
No	288	86 (30%)		146	35 (24%)	
No data	2	0 (0%)		1	0 (0%)	
Anti HER2 therapy (trastuzumab, lapatinib)			**0.012**			**0.027**
Yes	81	18 (22%)		37	5 (14%)	
No	311	115 (37%)		163	52 (32%)	
No data	1	0 (0%)		1	0 (0%)	
Chemotherapy			**0.022**			0.054
Mono-CHT	87	24 28%)		39	8 (21%)	
Poly-CHT	109	35 (32%)		64	18 (28%)	
Bevacizumab + CHT	93	44 (49%)		47	21 (45%)	
Other CHTs	41	9 (22%)		22	3 (14%)	
No CHT	62	21 (34%)		28	7 (25%)	
No data	1	0 (0%)		1	0 (0%)	
Radiological response after first cycle of chemotherapy			0.104			**0.011**
CR/PR/SD	162	52 (32%)		99	23 (23%)	
PD	93	40 (43%)		55	24 (44%)	
No data	138	41 (30%)		47	10 (21%)	

*Percentages of the respective row total for baseline and first-cycle data.

CHT, chemotherapy; CR, complete response; ER, estrogen receptor; HER2, human epidermal growth factor receptor 2; HR, hormone receptor; PD, progressive disease; PgR, progesterone receptor; PR, partial response; SD, stable disease; TNBC, triple negative breast cancer.

*P*-values were calculated for differences between CTC+ and CTC- groups using the Wilcoxon test or Fisher’s exact test, as appropriate. Bold *P* values indicate statistical significance.

### CTC status and survival

Follow-up data were available for 356 patients with a median [95% CI] follow-up of 26.0 [23.7–28.5] months for OS.

**Figure 2:**
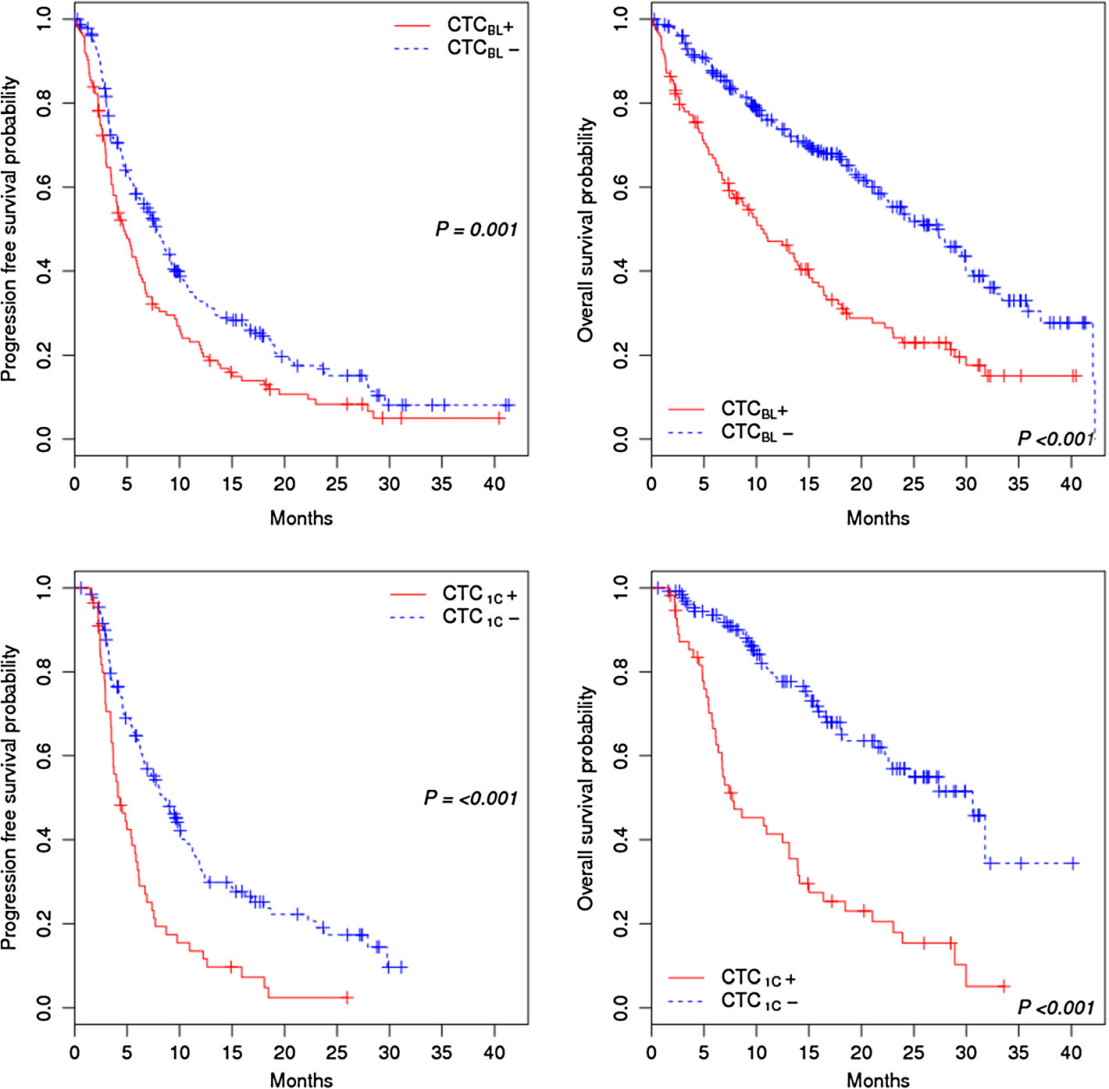
**Progression-free survival and overall survival by CTC status.** PFS (left) and OS (right) by CTC status at baseline (top) and after the first cycle of a new line of systemic therapy (bottom) in 356 patients with MBC.

Figure 2 shows Kaplan-Meier plots for PFS and OS by CTC status at baseline (CTC_BL_, top panels) and after the first cycle of a new line of systemic therapy (CTC_1C_, bottom panels). Median [95% CI] PFS and OS were significantly shorter in CTC_BL_+ than in CTC_BL_- patients (PFS: 4.7 [3.7–6.1] vs. 7.8 [6.4–9.2] months, *P* = 0.001; OS: 10.4 [7.9–15.0] vs. 27.2 [22.3–29.9] months, *P* < 0.001). Median [95% CI] PFS and OS were also significantly shorter in CTC_1C_+ than in CTC_1C_- patients (PFS: 4.3 [3.6–6.0] vs. 8.5 [6.6–10.4], *P* < 0.001; OS: 7.7 [6.4–13.9] vs. 30.6 [22.6–na], *P* < 0.001).

**Figure 3:**
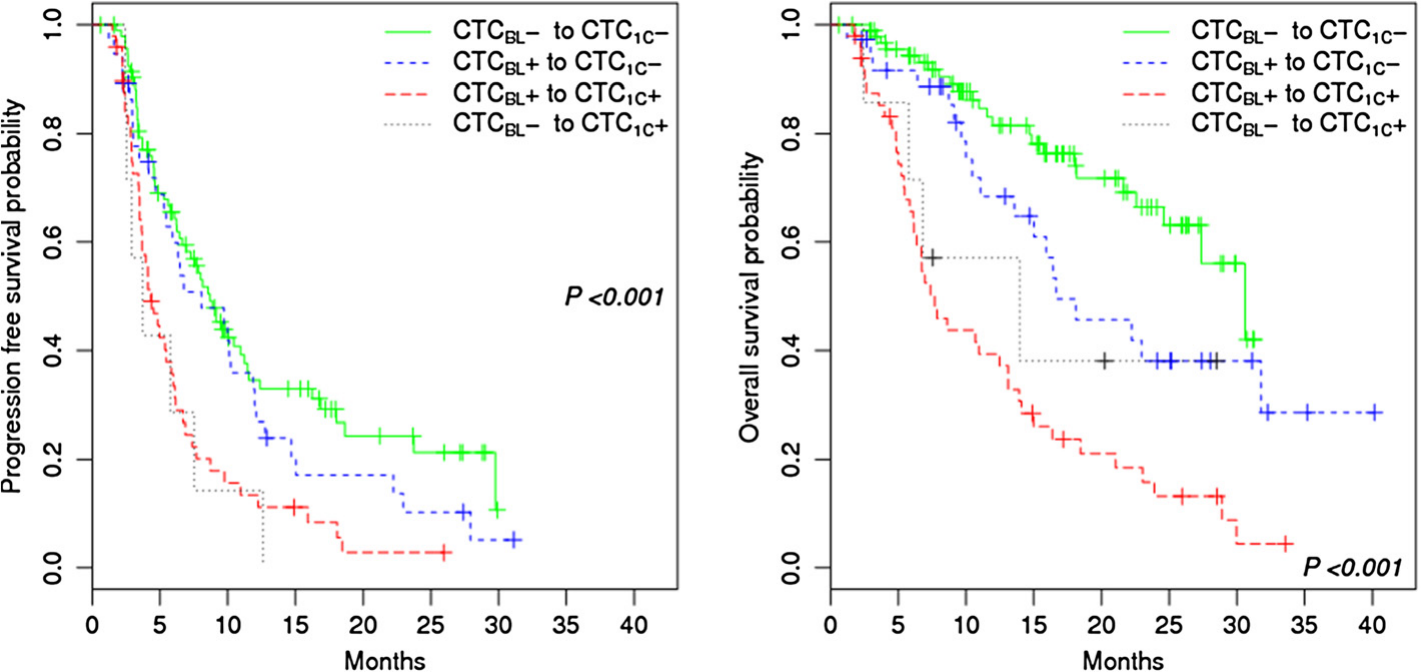
**Progression-free survival and overall survival by CTC**_**KIN**_**.** PFS (left) and OS (right) stratified by change in CTC status (CTC_KIN_) from baseline to completion of the first treatment cycle.

Figure 3 shows Kaplan-Meier plots for PFS and OS stratified by change in patients’ CTC status from baseline to completion of the first treatment cycle (CTC_KIN_). There were significant differences in PFS and OS, depending on CTC_KIN_ (*P* < 0.001 for PFS and OS). For PFS, we simplified to favorable and unfavorable CTC_KIN_, depending on CTC_1C_ status. PFS for patients with favorable CTC_KIN_ (i.e. CTC_BL_- to CTC_1C_- or CTC_BL_+ to CTC_1C_-) did not differ significantly (*P* = 0.251). Similarly, PFS for unfavorable CTC_KIN_ (i.e. CTC_BL_- or CTC_BL_+ to CTC_1C_+) also showed no significant difference (*P* = 0.665). Regarding OS, CTC_BL_ status also appeared important since patients with CTC_KIN_ from CTC_BL_- to CTC_1C_- lived significantly longer than those with CTC_KIN_ from CTC_BL_+ to CTC_1C_- (*P* = 0.049). OS times for unfavorable CTC_KIN_ did not differ significantly (*P* = 0.358). When conditioning on non-missing CTC_1C_ values, the median OS time was overestimated by 2.7 months for CTC_BL_+ and 3.4 months for CTC_BL_- patients. This provides a rough estimate of the effect of deaths before CTC_1C._ No CTC_1C_ status was obtained for 12/12, 8/10, and 5/13 patients who died during the first, second, and third month after study entry, respectively. No CTC_1C_ status was obtained for 26/40, 3/5, and 0/6 patients who were censored during the first, second, and third month after study entry, respectively. Table 2 summarizes the results for PFS, OS, and progression by CTC_KIN_.

**Table 2:** **CTC**_**KIN **_**and association with PFS, OS, and progression at 3-month radiological examination**

	**CTC**_**BL **_**(baseline)**		**CTC**_**1C **_**(after 1st cycle)**	**PFS (months)**	**OS (months)**	**Progression**
				**Median [95% CI]**	**Median [95% CI]**	**Numbers (percentage)**
**Favorable**	**Negative****(CTC**_**BL**_**-)**		**Negative****(CTC**_**1C**_**-)**	8.7 [6.6–11.5]	30.6 [27.4–na]	20/75 (27%)
	**Positive****(CTC**_**BL**_**+)**		**Negative****(CTC**_**1C**_**-)**	8.0 [5.5–12.1]	16.7 [13.6–na]	11/32 (34%)
**Unfavorable**	**Positive****(CTC**_**BL**_**+)**		**Positive****(CTC**_**1C**_**+)**	4.3 [3.6–6.1]	7.7 [6.1–13.1]	21/41 (51%)
	**Negative****(CTC**_**BL**_**-)**		**Positive****(CTC**_**1C**_**+)**	3.7 [2.5–na]	14.0 [5.7–na]	3/6 (50%)

na = not available.

### Response and survival

Survival depended significantly on the result of radiological assessment 3 months after inclusion as median [95% confidence interval (CI)] OS times were 29.9 [27.4–37.1] months for patients who achieved at least SD, and 13.6 [9.1–16.4] months for patients with PD (n = 356; *P* < 0.001).

### Multivariate regression analysis

Table 3 shows the result of multivariate regression analysis for PFS and OS using a Cox proportional hazards model including CTC_BL_, age at study entry, number of metastatic sites, site of metastasis, line of therapy, and molecular subtypes. Significant risk factors for progression were CTC_BL_+ status, third or higher line of therapy, and TNBC. Significant risk factors for death were CTC_BL_+, both visceral/local and bone metastases, third or higher line of therapy, and TNBC. The concordance index was 0.62 for the PFS Cox model and 0.71 for the OS Cox model.

**Table 3:** **Cox proportional hazards model with CTC**_**BL**_

	**PFS**			**OS**		
	**Hazard ratio**	**95% CI**	***P***	**Hazard ratio**	**95% CI**	***P***
**Baseline CTC status (CTC**_**BL**_**)**						
< 5 CTC (CTC_BL_-)	1.00			1.00		
≥ 5 CTC (CTC_BL_+)	1.55	1.19–2.01	**0.001**	2.79	2.04–5.63	**< 0.001**
***Age at inclusion***						
Per year	0.99	0.98–1.00	0.207	1.00	0.99–1.01	0.938
***Number of metastatic sites***						
One site	1.00			1.00		
Multiple sites	0.97	0.66–1.43	0.892	0.71	0.41–1.23	0.227
**Site of metastasis**						
Bone	1.00			1.00		
Visceral/local	0.98	0.67–1.46	0.939	1.75	1.00–3.01	0.052
Both	1.07	0.71–1.63	0.739	2.55	1.41–4.60	**0.002**
**Line of therapy**						
1	1.00			1.00		
2	1.35	0.93–1.95	0.113	1.45	0.92–2.28	0.112
≥ 3	1.91	1.40–2.59	**< 0.001**	2.01	1.37–2.96	**< 0.001**
**Molecular Subtypes**						
HR+/HER2-	1.00	°		1.00		
HER2+	1.10	0.79–1.52	0.577	1.09	0.72–1.63	0.695
TNBC	1.92	1.36–2.71	**< 0.001**	2.86	1.91–4.27	**< 0.001**

Bold *P* values indicate statistical significance.

HR, hormone receptor; HER2, human epidermal growth factor receptor 2; TNBC, triple negative breast cancer.

Table 4 shows the result of multivariate regression analysis for PFS and OS using a Cox proportional hazards model including CTC_KIN_. In this model, significant risk factors for both progression and death were CTC_BL_+ to CTC_1C_+ kinetics, line of therapy, and TNBC. The presence of both visceral/local and bone metastases was an additional significant risk factor for OS. The concordance index was 0.67 for the PFS and 0.80 for the OS Cox model.

**Table 4:** **Cox proportional hazards model with CTC**_**KIN**_

	**PFS**			**OS**		
	**Hazard ratio**	**95% CI**	***P***	**Hazard ratio**	**95% CI**	***P***
***CTC***_***KIN***_						
CTC_BL_- to CTC_1C_-	1.00			1.00		
CTC_BL_+ to CTC_1C_-	1.01	0.62–1.64	0.981	1.68	0.85–3.32	0.135
CTC_BL_+ to CTC_1C_+	2.17	1.39–3.37	**< 0.001**	5.58	3.06–10.15	**< 0.001**
CTC_BL_- to CTC_1C_+	2.17	0.91–5.14	0.079	2.56	0.76–8.00	0.134
***Age at inclusion***						
Per year	0.99	0.98–1.00	0.333	0.99	0.97–1.01	0.284
***Number of metastatic sites***						
One site	1.00			1.00		
Multiple sites	0.94	0.98–1.01	0.832	0.59	0.24–1.48	0.260
***Site of metastasis***						
Bone	1.00			1.00		
Visceral/local	1.10	0.59–2.04	0.768	2.12	0.82–5.49	0.124
Both	1.27	0.68–2.37	0.449	3.35	1.27–8.82	**0.014**
***Line of therapy***						
1	1.00			1.00		
2	1.66	1.00–2.75	**0.049**	2.01	1.02–3.99	**0.045**
≥ 3	2.49	1.58–3.94	**< 0.001**	2.49	1.32–4.65	**0.004**
*Molecular Subtypes*						
HR+/HER2-	1.00			1.00		
HER2+	1.34	0.81–2.19	0.252	1.37	0.64–2.91	0.418
TNBC	2.58	1.53–4.35	**< 0.001**	3.92	2.11–7.30	**< 0.001**

Bold *P* values indicate statistical significance.

HR, hormone receptor; HER2, human epidermal growth factor receptor 2; TNBC, triple negative breast cancer.

## Discussion

In recent years, several retrospective and a few prospective studies have demonstrated the strong and independent prognostic role of CTCs in MBC [1,2,4,9,11,15]. Using the FDA-cleared CellSearch™ system, detection of ≥ 5 CTCs/7.5 ml blood before starting a new line therapy is associated with decreased PFS and OS. In addition, CTCs provide an effective prognostic tool for early response prediction as survival is prolonged once counts ≥ 5 CTCs/7.5 ml blood convert to < 5 CTCs/7.5 ml, i.e. from CTC positive to CTC negative [4,7,9,16]. Thus, serial CTC enumeration promises to provide a fast and easy-to-perform tool for monitoring the efficacy of a given systemic treatment in MBC patients [7]. To address this directly in a clinical setting, the present large study analyzed the changes in CTC status, or CTC kinetics, occurring from baseline to completion of the first cycle of a new line of systemic therapy in patients with MBC. The data were then analyzed to prospectively determine the association of CTC status and first-cycle CTC status with treatment response, PFS, and OS.

Our data demonstrate that patients with favorable CTC kinetics, i.e. those whose CTC status after one cycle of therapy (CTC_1C_) was negative, were more likely to respond to therapy as determined by RECIST criteria than patients with persistently high CTC counts [5,8,16,17]. Furthermore, PFS was significantly longer in patients with a negative CTC_1C_ status than in those who were CTC positive after completing the first treatment cycle. This observation was independent of the CTC status at baseline, supporting the role of serial CTC enumeration as a means of assessing treatment response. Accordingly, multivariate analysis showed no impact of a positive baseline CTC status on PFS if CTC status turned negative after one cycle of treatment. Budd et al. found CTC assessment to be predictive of survival in both patients with and without radiological progression [5]. They also suggested that CTC assessment might have advantages over radiographic evaluation, including higher reproducibility due to lower interreader variability, useful results at an earlier time, and more robust prediction of survival [5]. Imaging studies, currently the gold standard surrogate for clinical benefit from systemic therapy, are usually not performed before completion of at least two or three cycles of therapy. Hence, CTC determination after one cycle might enable much earlier assessment of treatment response and thus spare patients the unnecessary side effects of ineffective but toxic treatments. Moreover, radiographic imaging is confounded by a considerable degree of intraobserver and interobserver variability, whereas CTC enumeration with the CellSearch™ system is highly standardized [18].

In the current study, the majority of patients (66%) were CTC negative at baseline. This is in contrast to a seminal analysis provided by Cristofanilli et al. [2], who reported 70% of the patients harboring ≥ 5 CTCs/7.5 ml blood. However, in our study, only 31% of patients received third- or higher-line therapy. Thus, the difference might be due to a selection bias.

Other explanations, however, are also conceivable. Despite the prognostic impact of CellSearch CTC in MBC, it has become clear that this technology has limitations. In particular, it is not capable of detecting the entire, highly heterogeneous population of CTCs as it involves EpCAM-based capturing methods [19]. Moreover, a recent retrospective study in 292 MBC patients reported that the probability of undetectable CTCs was increased in patients with negative hormone receptors, high tumor grade, triple-negative disease, and inflammatory breast cancer [20]. The authors suggested that these findings might reflect underestimation of CTCs by CellSearch due partly to CTCs undergoing epithelial-mesenchymal transition (EMT). An earlier study found that a major proportion of CTCs in the blood of MBC patients showed EMT and tumor stem cell characteristics and that such CTCs were associated with an inferior prognosis [21]. On the other hand, it has recently been demonstrated that not all patients with detectable CTCs have a poor prognosis, suggesting that further characterization of these cells might provide more information on their biologic significance. In this regard, Smerage et al. [22] used CellSearch to analyze CTC apoptosis and Bcl-2 expression and show that determination of these markers may have biological and clinical implications. This, therefore, might also offer a further explanation for the large proportion of CTC negative patients in the present study. Moreover, therapeutic regimens might also explain the high CTC negativity rate. A combination of e.g. trastuzumab and lapatinib might be more effective in HER2 positive patients and even stem cell-like cells might be eliminated by such a combination.

In our study, patients with a negative CTC status after the first cycle had a significantly prolonged OS if they were CTC negative at baseline. This observation is in line with results reported by Pierga et al. [9], showing that OS was better in patients with persistently low CTC counts (< 5 CTCs/7.5 ml blood) than in initially CTC positive patients with low CTC counts after one treatment cycle. In addition, it indicates that baseline CTC determination enables identification of more aggressive disease and thus may be valuable in making an early decision whether patients require more aggressive or less aggressive treatment [15]. Of note, the group of baseline positive patients in our study was significantly younger than the baseline negative patients at the time of study entry, although there was no significant difference with respect to age at initial diagnosis. This further supports the hypothesis that higher CTC counts may be suggestive of more aggressive disease in younger women.

Advantages of the CellSearch™ system include semi-automation and proven reproducibility, reliability, sensitivity, linearity, and accuracy [13]. However, it is important to bear in mind that 66% of MBC patients in our cohort had < 5 CTCs/7.5 ml blood at baseline. During the initial phase of the study, which comprised the first 100 patients, CTC status at follow-up was only assessed in patients who had been CTC positive at baseline. Due to the unexpectedly low CTC positivity at baseline, we decided also to evaluate initially CTC negative patients for CTC status at follow-up. However, only 7% of the patients who were CTC negative at baseline were found to be CTC positive after one cycle of treatment. Therefore, it seems that CTC counts, as measured by the CellSearch™ system, are useful as a tool for monitoring treatment efficacy only in patients who are CTC positive when they start a new line therapy, highlighting the need for additional, more sensitive methods of CTC detection. In addition, methods based on the detection of EpCAM, like the CellSearch™ system, might miss CTCs that have undergone epithelial-mesenchymal transition [23].

We found a strong relationship between treatment-associated CTC kinetics and outcome. Favorable CTC_KIN_ was associated with a significantly better disease control rate. In addition, patients with high baseline CTC counts ≥ 5 CTCs/7.5 ml blood that decreased to < 5 CTCs/7.5 ml blood after one cycle of treatment had a PFS similar to patients with baseline counts < 5 CTCs/7.5 ml [24]. In contrast, OS depends not only on the patient’s current CTC status, but also on her previous CTC history. For instance a patient with a CTC_1C_- status had a better prognosis if she was initially CTC_BL_- rather than CTC_BL_+. Thus, a patient’s CTC history might better reflect the overall aggressiveness and prognosis of her breast cancer than the current CTC status alone. Using a somewhat different, CTC count-based approach to classifying CTC kinetics, a recent study by Hartkopf et al. demonstrated that changes in CTC levels from baseline to completion of three treatment cycles also correlated with radiological response and were associated with survival [17]. Median OS was significantly longer in patients with decreasing CTC levels than in patients with increasing CTC counts.

Data from this and other studies [5,8,9,16,17] do not allow the distinction between breast cancers with unfavorable CTC kinetics that are resistant to the specific type of chemotherapy administered versus those that are resistant to chemotherapy in general. Ongoing prospective trials such as the Southwest Oncology Group (SWOG) protocol S0500 trial and the DETECT III trial will help to shed light on the utility and limitations of measuring CTCs to monitor response to treatment. The SWOG trial randomly assigns MBC patients with persistent CTC counts ≥ 5/7.5 ml blood at the follow-up visit to either continuation of their current therapy or switching to a different regime. DETECT III is a multicenter phase III trial comparing standard therapy +/- lapatinib in HER2 negative MBC patients but with HER2 positive CTCs.

The potential of CTC enumeration and characterization to serve as a “liquid real-time biopsy”, i.e. as a noninvasive means of predicting and monitoring response to treatment in metastatic disease, has recently been comprehensively discussed by Alix-Panabieres and Pantel [25]. Unsuccessful regimens could be abandoned early in favor of alternative regimens, thus sparing patients unnecessary toxicity [6-8]. Moreover, in the future real-time CTC enumeration during therapy should be complemented by additional markers, which enable the monitoring of those cells which possess the highest metastasis-inducing activity within the highly heterogeneous pool of EpCAM+ CTCs [4,26]. For ER+ luminal MBCs such metastasis-initiating cells have been functionally defined as EpCAM+/CD44+/MET+/CD47+ [4,26]. However, novel methods have yet to be developed to include these markers in routine clinical practice. Future studies are needed to investigate ways in which CTC enumeration can be combined with computer-assisted assessment of prognosis and adjuvant therapy planning based on various biomarkers [27-29] to further individualize and target the treatment of breast cancer, which remains the most frequent cancer in women in Germany and worldwide [30].

In summary, our study demonstrates that serial CTC monitoring is a versatile tool for predicting treatment outcome in MBC and a useful adjunct to standard diagnostic tests for tailoring therapy. The data presented here further support the hypothesis that the monitoring of CTCs is a promising source of biological information towards predicting the course of disease and its responsiveness to targeted agents, hence paving the way for individualized therapy [24,25,31,32].

## Conclusions

CTC status at baseline (CTC_BL_) and after one cycle of a new line of therapy (CTC_1C_) and CTC kinetics (CTC_KIN_, i.e. changes from CTC_BL_ to CTC_1C_) are highly predictive of outcome in MBC and significantly associated with PFS and OS.

Based on the findings of the present prospective study, we consider serial CTC monitoring a versatile tool for predicting treatment outcome in MBC and a useful adjunct to standard diagnostic tests in tailoring therapy.

## Abbreviations

CHT: Chemotherapy; CI: Confidence interval; CR: Complete response; CT: Computed tomography; CTC: Circulating tumor cell; DKFZ: German Cancer Research Center; EDTA: Ethylenediaminetetraacetic acid; EpCAM: Epithelial cell adhesion molecule; ER: Oestrogen receptor; HER-2: Human epidermal growth factor receptor-2; HR: Hormone receptor (estrogen and progesterone); HT: Hormonal therapy; MBC: Metastatic breast cancer; MRI: Magnetic resonance imaging; NCT: National Center for Tumor Diseases; OS: Overall survival; PD: Progressive disease; PFS: Progression-free survival; PgR: Progesterone receptor; PR: Partial response; SD: Stable disease; RECIST: Response Evaluation Criteria in Solid Tumors; STD: Standard deviation; SWOG: Southwest Oncology Group; TNBC: Triple negative breast cancer.

## Competing interests

The authors declare that they have no competing interests.

## Authors’ contributions

MW, AS, BB, KP, and AT conceived of the study and designed it. MW and AS supervised the study. SR and KP developed the methodology. MW, CM, CD, AS, ADH, SR, JN, DM, MRS, SS, IB, BB, FM, JH, CS, KP, and AT participated in patient recruitment, patient management, clinical data collection, and sample collection and analysis. BS, JN, DM, and SS participated in organizing or reporting the data and constructing databases, and conducted data management. BS performed the statistical analysis. MW, AS, IB, BS, JH, CS, MRS, BB, SR, and AT participated in data analysis and interpretation. MW, AS, and BS drafted the manuscript. ADH, CM, SR, JN, DM, MRS, SS, CD, IB, BB, FM, JH, CS, KP, and AT revised the draft manuscript for important intellectual input. AT and AS contributed equally as joint senior authors. All authors read and approved the final manuscript.

## Authors’ information

Andreas Trumpp and Andreas Schneeweiss are joint senior authors.

## Pre-publication history

The pre-publication history for this paper can be accessed here:

http://www.biomedcentral.com/1471-2407/14/512/prepub
